# MicroRNAs in Cutaneous Melanoma: Biological Roles, Clinical Utility, and Future Directions

**DOI:** 10.3390/medsci14060598

**Published:** 2026-09-23

**Authors:** Adrian-Ilie Pașcu, Maximilian-Vlad Muntean, Claudia Diana Gherman, Vlad Alexandru Gâta, Simona Corina Şenila, Salomea-Ruth Halmágyi, Loredana Ungureanu

**Affiliations:** 1 Department of Dermatology, “Iuliu Hațieganu” University of Medicine and Pharmacy, 400006 Cluj-Napoca, Romania; adr.pascu.97@gmail.com (A.-I.P.); corina.senila@umfcluj.ro (S.C.Ş.); h_salomea@yahoo.com (S.-R.H.); loredana.ungureanu@umfcluj.ro (L.U.); 2 Department of Plastic Surgery, “Iuliu Hațieganu” University of Medicine and Pharmacy, 400006 Cluj-Napoca, Romania; 3 Department of Plastic Surgery, The Oncology Institute “Prof. Dr. Ion Chiricuţă”, 400015 Cluj-Napoca, Romania; 4 Department of Surgery-Practical Abilities, “Iuliu Hațieganu” University of Medicine and Pharmacy, 400006 Cluj-Napoca, Romania; gherman.claudia@umfcluj.ro; 5 Department of General Surgery No.2, Emergency County Hospital, 400006 Cluj-Napoca, Romania; 6 Department of Oncological Surgery, “Iuliu Hațieganu” University of Medicine and Pharmacy, 400006 Cluj-Napoca, Romania; vlad.gata@umfcluj.ro; 7 Department of Oncological Surgery, The Oncology Institute “Prof. Dr. Ion Chiricuţă”, 400015 Cluj-Napoca, Romania; 8 Department of Dermatology, Emergency County Hospital, 400006 Cluj-Napoca, Romania

**Keywords:** circulating microRNAs, cutaneous melanoma, liquid biopsy, biomarkers, diagnosis, prognosis, miRNA signature

## Abstract

Cutaneous melanoma remains a malignancy with substantial metastatic potential, and conventional diagnostic and surveillance tools such as biopsy and imaging have recognized limitations in sensitivity, specificity, and invasiveness. Circulating microRNAs (miRNAs) have emerged as promising non-invasive biomarkers because of their stability in body fluids and their disease-specific expression patterns. This narrative review summarizes current evidence on the biological roles of miRNAs in melanoma, acting as either oncogenes or tumor suppressors, and their potential clinical utility across diagnosis, prognosis, treatment monitoring, and therapeutic targeting. Individual circulating miRNAs (e.g., miR-21, miR-221, miR-206, miR-125b) and multi-miRNA signatures (e.g., MEL12, MEL38, MELmiR-7) have shown associations with tumor stage, recurrence, and survival, and several panels have demonstrated high diagnostic sensitivity and specificity in liquid biopsy assays. miRNAs also participate in drug resistance and immune modulation, suggesting relevance to immune checkpoint inhibitor therapy and potential as therapeutic targets. However, clinical translation remains limited by the lack of standardized isolation and quantification protocols, tumor and patient heterogeneity, and the predominantly associative nature of current evidence. Continued methodological standardization, larger validation cohorts, and integration with other omics platforms will be needed before circulating miRNAs can be incorporated into routine melanoma management.

## 1. Introduction

Cutaneous melanoma is a malignant tumor originating from melanocytes and is characterized by rapid metastatic spread through both hematogenous and lymphatic routes. It accounts for approximately 5% of newly diagnosed cancer cases annually, and its global incidence continues to rise [1]. In superficial spreading melanoma, growth initially occurs in a horizontal pattern and later becomes vertical, at which point most metastases arise. The horizontal growth phase is particularly important for early diagnosis. In nodular melanoma, however, growth is predominantly vertical from the outset, which should be considered carefully in early detection [1].

Melanomas most commonly occur on the lower limbs in females and on the trunk in males. Approximately 30% of melanomas arise from pre-existing melanocytic lesions, whereas 70% develop de novo, without a prior lesion [1]. In the AJCC staging system, melanoma patients are classified into three groups: localized disease (stage I–II, without evidence of metastasis), regional disease (stage III, with spread to regional lymph nodes), and distant disease (stage IV, with metastatic involvement of distant sites) [2].

Follow-up care for treated melanoma patients typically involves regular monitoring to detect recurrence or the development of new primary melanomas. Such follow-up is essential for the early identification of both recurrence and second primary disease. However, no international consensus exists regarding the optimal surveillance schedule or the specific tests to be used. The exact timing and methods of follow-up vary depending on melanoma stage, the treatment received, and the patient’s overall health status [1,3,4].

The current NCCN Cutaneous Melanoma guideline (Version 2.2026) [2] reflects several practice changes that are relevant to how patients in this review are managed and monitored; most notably, neoadjuvant systemic therapy (pembrolizumab, or ipilimumab plus nivolumab) is now the preferred approach for resectable nodal disease rather than upfront surgery, and risk-stratified follow-up intervals continue to be revised as new evidence emerges. Regular clinical examinations by healthcare professionals, together with self-examinations by patients, are essential components of melanoma surveillance. Many recurrences are first detected by patients themselves, underscoring the importance of patient education and self-monitoring [3,5,6]. Imaging techniques such as CT, PET-CT, and MRI are often used, especially in higher-stage melanoma, typically stage IIC and above. However, the frequency and necessity of these investigations differ considerably among guidelines [7,8,9,10]. Lymph node sonography is a promising tool for detecting recurrences, particularly in stage IB and higher disease, although its impact on survival has not yet been fully established [4,8,9]. Current surveillance recommendations for cutaneous melanoma are risk-stratified according to disease stage at diagnosis, as outlined in the NCCN Clinical Practice Guidelines [2]. For patients with stage 0–IA disease, follow-up typically consists of routine history and physical examination, including a thorough skin and lymph node check, every 6–12 months for the first several years, with imaging reserved for symptom-directed evaluation only. Patients with stage IB–IIA disease follow a similar clinical-examination schedule but are seen somewhat more frequently, generally every 3–6 months for the first 2–3 years before intervals lengthen. For higher-risk stage IIB–IV disease, surveillance intensifies further, with clinical examinations every 3–6 months for the first 2–3 years and periodic imaging (such as CT, PET-CT, or MRI) and serum LDH considered on a risk-adjusted basis to detect recurrence or metastatic disease earlier than physical examination alone would allow. Across all risk groups, patient education on monthly self-skin examination is emphasized as an adjunct to clinician-led surveillance, and follow-up generally continues for at least 5 years, with lifelong annual skin examinations recommended given the persistent risk of second primary melanomas.

Circulating biomarkers in melanoma have attracted increasing attention because they may provide non-invasive, real-time information about disease status, thereby improving diagnosis, prognosis, and treatment monitoring. These biomarkers include circulating tumor cells (CTCs), exosomes, microRNAs, and circulating tumor DNA (ctDNA), each of which presents unique advantages and limitations [11]. Early detection and accurate prognostication are critical for improving patient outcomes, yet conventional diagnostic tools such as biopsy and imaging have limitations in terms of sensitivity, specificity, and invasiveness. In this context, circulating microRNAs (miRNAs) have emerged as promising non-invasive biomarkers [12].

This review aims to provide a comprehensive overview of current research on circulating miRNAs in cutaneous melanoma. By examining the diagnostic, prognostic, and therapeutic potential of miRNAs, as well as the challenges and future directions in the field, this review highlights the transformative potential of these biomarkers in melanoma management.

Literature Search Strategy and Scope: This narrative review is based on a search of PubMed/MEDLINE, supplemented by Google Scholar to capture conference abstracts and titles not yet indexed in MEDLINE, covering literature published from January 2008 through July 2026. Search terms combined “circulating microRNA” or “circulating miRNA” with “cutaneous melanoma,” further combined with “liquid biopsy,” “biomarker,” “diagnosis,” “prognosis,” “monitoring,” “drug resistance,” and “immunotherapy” to capture the clinical applications covered in this review. Reference lists of retrieved articles were also manually screened for additional relevant studies, which is how several conference abstracts and recently published articles outside the core search terms were identified. General melanoma staging, surveillance, and clinical-guideline references predating 2008 were incorporated from established clinical-management literature rather than from this miRNA-focused search, and standard dermatology reference texts (e.g., Fitzpatrick’s Color Atlas and Synopsis of Clinical Dermatology) were consulted directly for introductory background rather than retrieved through the database search. Eligible studies included those reporting circulating (serum, plasma, whole blood, or extracellular vesicle) microRNA measurements in cutaneous melanoma; a smaller number of tissue-based microRNA studies are also discussed where they provide necessary biological context and are explicitly labeled as tissue-derived throughout this review to avoid the implication that tissue expression necessarily predicts circulating levels. As this is a narrative rather than a systematic review, no formal quality-of-evidence grading (e.g., GRADE) was applied. Where relevant, we note whether evidence derives from small, single-center, or preclinical studies versus larger validated cohorts. The great majority of the circulating-miRNA studies discussed enrolled patients with cutaneous melanoma broadly, without stratifying analyses by histological subtype (e.g., superficial spreading, nodular, lentigo maligna, acral lentiginous). Subtype is generally reported only as a baseline cohort characteristic rather than analyzed as a variable in these studies. The near-absence of subtype-stratified circulating-miRNA data, including for acral melanoma specifically, is discussed further as a limitation in “Challenges and Future Directions”.

## 2. General Features of miRNAs

In the mammalian genome, approximately 60–70% of total DNA is transcribed into RNA, whereas only about 2% encodes proteins. Over the past 25 years, accumulating evidence has challenged the traditional view that many genomic sequences are non-functional, revealing instead their important roles in gene regulation. Various classes of non-coding RNAs, including both long and small molecules, have been shown to influence chromatin function and transcription in eukaryotic cells. Among these, microRNAs (miRNAs) are a well-conserved class of small non-coding RNAs, typically 19–25 nucleotides in length, and their biogenesis is tightly regulated (Figure 1) [13]. They play an essential role in gene regulation and are frequently dysregulated in cancer, including melanoma. miRNAs are 19–25-nucleotide single-stranded non-coding RNAs that regulate gene expression by targeting the 3′ untranslated region of nearly 30% of human genes. Their 5′ seed region, comprising nucleotides 2 to 8, is critical for target recognition and allows miRNAs to function as either oncogenes or tumor suppressors. They exert their effects at post-transcriptional and translational levels by promoting mRNA degradation or inhibiting translation [14]. Their stability in body fluids and association with disease-specific states make them attractive candidates for diagnostics, prognostics, and therapy monitoring [15].

miRNAs play a crucial role in phenotype regulation. Although miRBase (release 22.1, October 2018 [16]) lists 2656 mature human miRNAs from 1917 precursors, the manually curated MirGeneDB 2.1 [17] retains only 556 bona fide human miRNA genes, highlighting ongoing debate about how many genuine human miRNAs exist. miRNA biogenesis begins in the nucleus, where RNA polymerase II transcribes a long primary miRNA (pri-miRNA) precursor consisting of several hundred nucleotides. This pri-miRNA, which adopts a stem–loop structure, is processed by the nuclear ribonuclease DROSHA, a member of the RNase III family, together with the double-stranded RNA-binding protein DGCR8, into a shorter precursor known as pre-miRNA, which is approximately 70 nucleotides long. Pre-miRNA is then transported into the cytoplasm via exportin-5/Ran-GTP, where it undergoes further cleavage by the ribonuclease DICER, another RNase III enzyme, together with the transactivation-responsive RNA-binding protein TRBP. This step generates a double-stranded miRNA duplex consisting of a guide strand (miRNA-5p) and a passenger strand (miRNA-3p), each approximately 21 nucleotides in length. Typically, only the guide strand (miRNA-5p) is incorporated into the miRNA-induced silencing complex (miRISC), where it functions as the mature miRNA. The passenger strand (miRNA-3p), formerly known as miRNA*, is usually degraded, although it can also be functional in some contexts, and the guide strand is not always the dominant regulatory molecule. miRISC regulates gene expression by binding to target mRNA, either fully or partially. In cases of perfect complementarity, miRISC induces mRNA degradation, whereas partial complementarity results in translational repression or mRNA deadenylation. Notably, miRNAs can also modulate gene expression at the transcriptional level by directly interacting with DNA. In addition, they may activate gene expression by targeting regulatory sequences. Beyond this canonical processing pathway, miRNA abundance is itself tightly regulated at multiple additional levels: transcription of miRNA-encoding genes is controlled by oncogenic and tumor-suppressive transcription factors in a manner analogous to protein-coding genes; miRNA loci can also be subject to epigenetic silencing through promoter hypermethylation; and the efficiency of Drosha/DGCR8 and Dicer/TRBP processing is itself modulated by RNA-binding proteins, providing a further layer of post-transcriptional control over mature miRNA output [18].

Abnormal expression of miRNA-encoding genes in malignant versus normal cells may result from their location in cancer-associated genomic regions, epigenetic mechanisms, and/or alterations in the miRNA-processing machinery. A few decades ago, alterations in oncogenes and tumor suppressor genes were considered the primary causes of tumorigenesis. The discovery of small non-coding RNAs has revealed a greater complexity in the mechanisms underlying cancer development and progression.

While approximately 300 miRNAs have been shown to play a role in carcinogenesis, more than 2000 miRNAs have been found to be differentially expressed in human cancers, and evidence suggests that different cancer types can be distinguished by miRNA profiling [17,19]. In cutaneous melanoma, increasing evidence supports an important role for specific miRNAs as oncogenes or tumor suppressor genes, as well as in other aspects of melanoma biology [20]. The diagnostic and prognostic potential of miRNA profiling in solid tissue samples from primary or metastatic lesions emerged alongside progress in understanding melanoma biology, but it has still not been incorporated into routine clinical practice [21]. A clinically important perspective has instead emerged through the discovery that circulating miRNAs may reflect different tumor types and indicate tumor stage and treatment response. Indeed, research has shown that quantitative analysis of cell-free miRNAs may assist clinical decision-making in cutaneous melanoma in terms of diagnosis, staging, recurrence prediction, and prognosis.

## 3. MicroRNAs as Biomarkers in Melanoma

MicroRNAs play a significant role in the development and progression of various cancers, including cutaneous melanoma. They may function as oncogenes or tumor suppressor genes (Figure 2), influencing cancer cell proliferation, invasion, and metastasis. In cutaneous melanoma, specific miRNAs have been identified as key regulators of these processes. miRNAs can act as oncogenes (oncomiRs) or tumor suppressors (anti-oncomiRs) in melanoma [20]. Oncogenic miRNAs are often upregulated, thereby promoting cancer progression, whereas tumor suppressor miRNAs are downregulated, leading to uncontrolled cell growth. Because of their stability in body fluids, miRNAs may serve as non-invasive diagnostic and prognostic biomarkers for melanoma progression and treatment response. They exhibit distinct expression profiles at different disease stages, which may aid in disease monitoring.

In addition, miRNAs are involved in drug resistance and therapy response in melanoma, including resistance to targeted therapies and immunotherapies [21]. They modulate immune responses and can influence the effectiveness of treatments such as immune checkpoint inhibitors. miRNAs also play a significant role in drug resistance mechanisms, suggesting that modulating their expression could help overcome chemoresistance in melanoma treatment. Their involvement in immune regulation may affect the efficacy of PD-1 and CTLA-4 inhibitors. Combining miRNAs with immune checkpoint blockade is being explored as a strategy to enhance therapeutic outcomes [19,22,23,24].

The dysregulation of miRNAs contributes to melanoma progression by affecting cell cycle regulation, apoptosis, and metastasis. Exosome-derived miRNAs, in particular, are involved in melanoma progression by facilitating invasion, metastasis, and immune escape, making them potential targets for therapeutic intervention [25]. The hsa-miR-193a-3p and -5p miRNAs have been identified as tumor suppressors in cutaneous melanoma. Their overexpression reduces cell viability and affects genes involved in proliferation and apoptosis, suggesting possible therapeutic applications [26]. Similarly, miR-153-3p has been identified as a tumor suppressor, inhibiting cell proliferation and invasion by targeting genes such as SNAI1 [27]. A review in Cells [28] highlighted the dual roles of miRNAs in melanoma, with some acting as oncogenes, such as miR-21, and others functioning as tumor suppressors, such as miR-34a. This duality reflects the complexity of miRNA regulation and emphasizes the need for comprehensive profiling to predict disease outcomes accurately.

To place melanoma’s circulating miRNA biomarkers in context, Table 1 compares them with representative, well-validated circulating miRNA biomarkers reported in other major solid tumors. miR-21 upregulation, for example, is a broadly shared, non-specific oncogenic circulating signal reported across at least 15 different solid tumor types (pooled sensitivity 78%, specificity 82%, AUC 0.87 across 36 studies [29]), indicating that melanoma’s clinically useful signal derives predominantly from multi-miRNA panels developed and validated specifically in melanoma cohorts (e.g., MEL38, MELmiR-7, MEL12) rather than from any single circulating miRNA.

## 4. Diagnosis, Prognosis, and Monitoring Biomarkers

MicroRNAs in serum are emerging as important biomarkers for melanoma, with potential applications in diagnosis, prognosis, and disease monitoring. Several studies have identified specific miRNAs that may serve as biomarkers for melanoma diagnosis and prognosis. Elevated levels of miR-221 and miR-21 in serum are associated with melanoma. These levels are significantly higher in patients with stage I–IV melanoma compared with those with melanoma in situ, correlating with tumor thickness and potentially serving as prognostic markers [34]. Decreased serum levels of miR-206 are associated with poor prognosis, more advanced clinical stage, and lower survival rates in melanoma patients, suggesting its utility as a non-invasive prognostic biomarker [35]. Lower levels of miR-125b in serum exosomes are linked with advanced melanoma, indicating its potential to reflect tumor-related dysregulation [36]. Similarly, miR-150-5p and miR-155-5p have been identified as potential diagnostic markers for stage IV melanoma, showing significant differences in expression between patients and healthy individuals, which supports their diagnostic value [21]. High baseline levels of miR-155-5p, miR-424-5p, and miR-320a are associated with better progression-free and overall survival in patients treated with immune checkpoint inhibitors, indicating their potential as predictive biomarkers [37]. Although miR-16 levels are reduced in melanoma patients compared with controls, they do not differ significantly between melanoma patients and healthy donors in some studies. Nevertheless, miR-16 is frequently used as a normalizer in miRNA studies [38,39]. Reports on serum/plasma miR-16 in melanoma have been inconsistent, and this variability is itself informative about miR-16′s reliability as a circulating biomarker in this setting. Friedman et al. [40] specifically evaluated the suitability of miR-16 as a normalizer for serum miRNA studies in melanoma and found no significant overall difference between melanoma patients and healthy controls (*p* = 0.37), although a significant difference was observed across melanoma stages (*p* = 0.015); formal equivalence testing across group pairings was not met, leading the authors to conclude that miR-16 is not a stable reference gene in this setting. A likely contributing technical factor is hemolysis: miR-16, together with miR-451, is highly abundant in red blood cells, and even mild, visually undetectable hemolysis during sample processing can inflate plasma miR-16 levels by up to 8-fold independent of disease status [41]. Mumford et al. [42] explicitly linked this pre-analytical confound to the melanoma miR-16 literature, noting that several published studies, including some cited in the present review, did not report exclusion of hemolyzed samples. Taken together, the inconsistent significance of miR-16 across studies most likely reflects a combination of stage-dependent expression and unaddressed hemolysis-related pre-analytical variability, rather than a true absence of biological difference between melanoma patients and controls.

Several studies have also investigated circulating miRNAs as diagnostic biomarkers for melanoma. A meta-analysis published in BMC Cancer in 2023 evaluated the diagnostic value of miRNAs and found high sensitivity and specificity. miRNAs such as miR-221-3p and miR-222-3p were significantly upregulated in melanoma patients compared with healthy controls [12]. These miRNAs are thought to exert oncogenic effects by targeting tumor suppressor genes and promoting melanoma progression. Another study published in the *British Journal of Dermatology* in 2023 validated a liquid biopsy assay based on circulating miRNAs. Van Laar et al. [15] showed that a 38-miRNA diagnostic signature distinguished melanoma patients from non-melanoma controls, with 93% sensitivity and 98% specificity and an AUC of 0.98. They also developed a 12-miRNA prognostic signature (MEL12) that effectively stratified patients into different risk groups and correlated with clinical staging and sentinel lymph node biopsy status. Patients classified as high risk by MEL12 were approximately three times more likely to have melanoma detected in their sentinel lymph nodes compared to low-risk patients. This assay, which included a panel of miRNAs, distinguished melanoma patients from healthy individuals with high accuracy. These findings support the potential of miRNA-based liquid biopsies as non-invasive diagnostic tools, particularly in early-stage melanoma, when traditional diagnostic methods may be less effective. Meta-analyses have likewise supported the diagnostic value of miRNAs, with pooled sensitivity and specificity values indicating their reliability as biomarkers [12].

Circulating miRNAs are considered ideal candidates for early melanoma detection because of their stability in blood and their dysregulation in cancer patients. However, the lack of standardized detection methods has hindered the identification of specific miRNA panels for early diagnosis.

Beyond diagnosis, circulating miRNAs also hold promise as prognostic biomarkers. A study published in *Anticancer Research* [39] found that higher preoperative plasma levels of miR-99a, miR-320, miR-1908, miR-494, and miR-4487 were significantly associated with a shorter disease-free interval, and that miR-99a, miR-221, miR-320, miR-494, miR-1260, and miR-1908 were significantly associated with worse overall survival in a prospective cohort of 22 patients with stage I–III melanoma. Separately, in a larger cohort of 283 patients, Fleming et al. identified a four-miRNA signature (miR-150, miR-30d, miR-15b, and miR-425) that separated patients by recurrence-free and overall survival, improving on AJCC stage alone [43]. These findings suggest the potential utility of prognostic circulating miRNA signatures in risk stratification and treatment planning. A specific circulating miRNA profile, comprising miR-1246 and miR-185, was identified as a biomarker of metastatic melanoma by Armand-Labit et al. [44], who validated it through a screening phase (14 metastatic melanoma patients vs. 5 controls, microarray), a training cohort (29 patients vs. 16 controls), and an independent validation cohort (31 patients vs. 43 controls, RT-qPCR), achieving 90.5% sensitivity and 89.1% specificity for distinguishing melanoma patients from controls. This suggests their potential use as non-invasive monitoring biomarkers.

A study presented at the American Association for Cancer Research (AACR) Annual Meeting in 2024 by Durante et al. [45] evaluated circulating miRNAs as predictive and prognostic biomarkers in advanced melanoma patients treated with immune checkpoint inhibitors (ICIs). Researchers collected blood samples from these patients and analyzed the absolute levels of miR-155-5p, miR-320a, and miR-424-5p using droplet digital PCR. They found that higher baseline levels of these miRNAs were associated with longer progression-free survival (PFS) and overall survival (OS). Specifically, in patients receiving anti-PD-1 therapy as first-line treatment, elevated baseline levels of miR-155-5p were correlated with better therapeutic responses, suggesting predictive value. In addition, baseline miR-155-5p levels were linked to certain CD4+ T-cell subpopulations, indicating a possible role in immune modulation. These findings suggest that baseline circulating miRNAs could serve as valuable biomarkers for prognosis and therapeutic outcomes in advanced melanoma patients undergoing immunotherapy [45].

Research by Segura et al. [46] has also shown that miRNA signatures may improve melanoma stratification beyond traditional staging methods. One study found a six-miRNA signature comprising miR-150, miR-342-3p, miR-455-3p, miR-145, miR-155, and miR-497, which was tested in 59 metastatic and primary melanoma samples plus congenital nevi and described as an independent predictor of post-recurrence survival in metastatic melanoma.

## 5. Clinical Utility Across Melanoma Care

This section critically evaluates whether circulating miRNAs provide meaningful added value over currently established methods at each stage of melanoma management and explicitly identifies where such evidence does not yet exist. Table 2 summarizes this assessment across nine clinical domains.

## 6. Therapeutic and Drug Resistance Markers

The role of miRNAs extends beyond diagnosis and prognosis to therapeutic applications. Modulating miRNA expression through synthetic mimics or inhibitors has shown promise in preclinical studies. For example, restoring the expression of tumor-suppressive miRNAs or inhibiting oncogenic miRNAs can disrupt critical pathways involved in melanoma progression, such as the PI3K/AKT and MAPK signaling cascades. miRNAs have emerged as important regulators in melanoma, influencing pathways involved in tumor progression, including the PI3K/AKT pathway and the CDK4/CDKN2A axis [55]. They are being explored as biomarkers for early detection and as therapeutic targets because of their ability to regulate genes associated with melanoma, including NRAS and c-KIT [14,55]. However, the clinical translation of miRNA-based therapies faces significant challenges. Issues such as off-target effects, delivery efficiency, and in vivo stability need to be addressed. Nevertheless, ongoing research into nanoparticle-based delivery systems and targeted therapies is paving the way for future clinical applications [56].

Circulating miRNAs have been explored as non-invasive biomarkers for monitoring treatment response and disease progression. Stark et al. [57] showed that the MELmiR-7 panel detected melanoma with 93% sensitivity and ≥82% specificity and that it characterized overall survival in melanoma patients better than serum LDH or S100B. In addition, baseline levels of miR-155-5p and other miRNAs have been associated with progression-free and overall survival in patients treated with immune checkpoint inhibitors [21].

Certain miRNAs, such as hsa-miR-150-5p and hsa-miR-155-5p, have been identified as important biomarkers for advanced melanoma, showing distinct expression profiles in patients compared with healthy controls. These miRNAs are also present in melanoma cell lines, providing a model for testing anti-melanoma therapies [23,55]. miRNAs play a significant role in drug resistance mechanisms, suggesting that their modulation could help overcome chemoresistance in melanoma treatment [58]. They are also involved in regulating immune responses, affecting the efficacy of immunotherapies such as PD-1 and CTLA-4 inhibitors. Combining miRNAs with immune checkpoint inhibitors is being explored as a strategy to enhance therapeutic outcomes [45,56].

## 7. Challenges and Future Directions

While the potential of miRNAs as biomarkers is promising, several challenges remain in standardizing analytical methods and validating findings across larger cohorts. Differences in miRNA expression profiles between studies highlight the need for further research to establish consistent and reliable biomarkers for clinical use.

First, the lack of standardized protocols for miRNA isolation, quantification, and analysis limits the reproducibility of results across studies. Different platforms, such as qRT-PCR, microarrays, and next-generation sequencing, yield variable results, complicating data interpretation [59]. Variability in pre-analytical and analytical methods creates difficulties in the consistent quantification of circulating miRNAs and contributes to conflicting findings across studies [60]. Cost and accessibility present a further practical barrier: comprehensive multi-miRNA panel assays remain expensive relative to standard clinical tests and are not widely available outside specialized or research laboratories, which may limit near-term adoption even where diagnostic performance is favorable.

Second, melanoma heterogeneity adds another layer of complexity. The molecular profiles of primary tumors, metastases, and circulating tumor cells may differ, leading to variations in circulating miRNA levels [61]. Furthermore, the influence of comorbidities, medications, and environmental factors on miRNA expression remains poorly understood.

Advances in bioinformatics and machine learning are enabling the development of predictive models based on miRNA signatures. These models may be used to personalize treatment strategies and monitor therapy response in real time. In addition, collaboration between academia, industry, and regulatory agencies will be essential for translating research findings into clinical practice. The field of circulating miRNA research in melanoma is evolving rapidly, with several emerging trends and opportunities for future investigation. Integrating miRNA profiling with other omics technologies, such as proteomics and metabolomics, could provide a more comprehensive understanding of melanoma biology. Multi-analyte liquid biopsies that combine miRNAs with other biomarkers, such as circulating tumor DNA or exosomal proteins, may offer superior diagnostic and prognostic accuracy.

Finally, although many studies report associations between miRNA levels and melanoma outcomes, establishing causality remains a challenge. Functional studies are needed to elucidate the precise roles of miRNAs in melanoma pathogenesis and progression. Beyond diagnostics, miRNAs may serve as therapeutic targets because of their role in tumorigenesis [62]. Research is ongoing to explore their potential in personalized treatment strategies for skin cancer. Developing miRNA-based therapeutics requires a deeper understanding of miRNA dysregulation during malignant transformation and of miRNA-associated proteins. Strategies include designing miRNA mimics or inhibitors and using nanotechnology for targeted delivery, although challenges such as delivery efficiency and off-target effects remain [56,63].

In conclusion, circulating miRNAs hold significant promise as biomarkers for melanoma prognosis and diagnosis. However, overcoming methodological challenges and achieving clinical validation are essential steps toward their integration into routine clinical practice.

## 8. Conclusions

Circulating miRNAs represent a promising frontier in the management of cutaneous melanoma. Their potential as non-invasive biomarkers for diagnosis, prognosis, and therapy monitoring is supported by a growing body of evidence, which is summarized in Table 3. However, significant challenges remain, including standardization of methodologies, understanding biological variability, and addressing technical limitations. By addressing these challenges and leveraging advances in technology and bioinformatics, circulating miRNAs could transform melanoma care. Continued research and collaboration will be essential to unlock their full potential and integrate them into routine clinical practice. This literature review underscores the importance of circulating miRNAs in melanoma and highlights the need for further investigation to realize their clinical utility. In conclusion, miRNAs represent a promising avenue for melanoma treatment, offering potential as both biomarkers and therapeutic targets. Ongoing research aims to refine miRNA-based strategies to improve diagnosis, overcome drug resistance, and enhance the effectiveness of existing therapies.

## Figures and Tables

**Figure 1:**
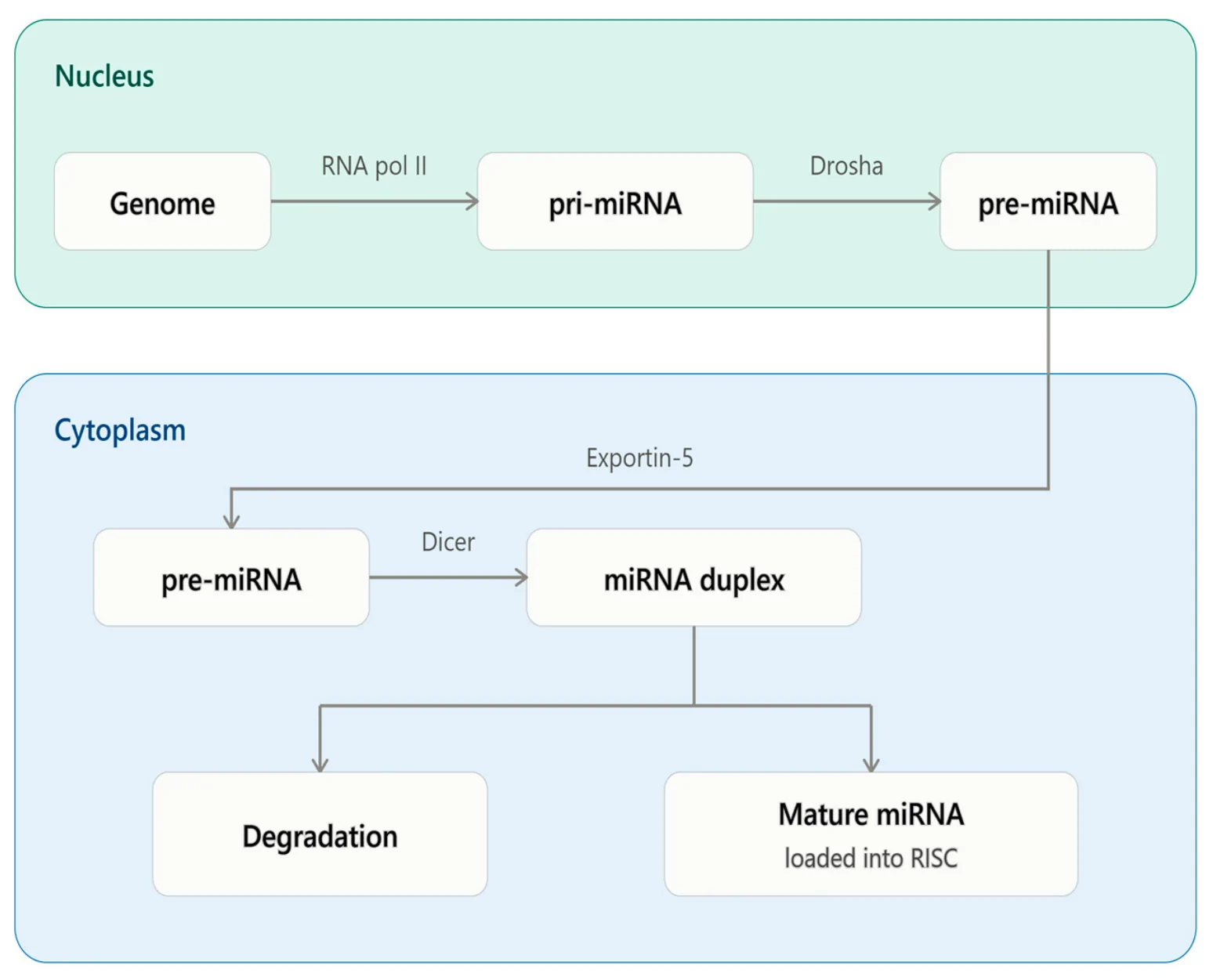
Pathway of miRNA biogenesis (created with Claude Sonnet 5 based on [13]).

**Figure 2:**
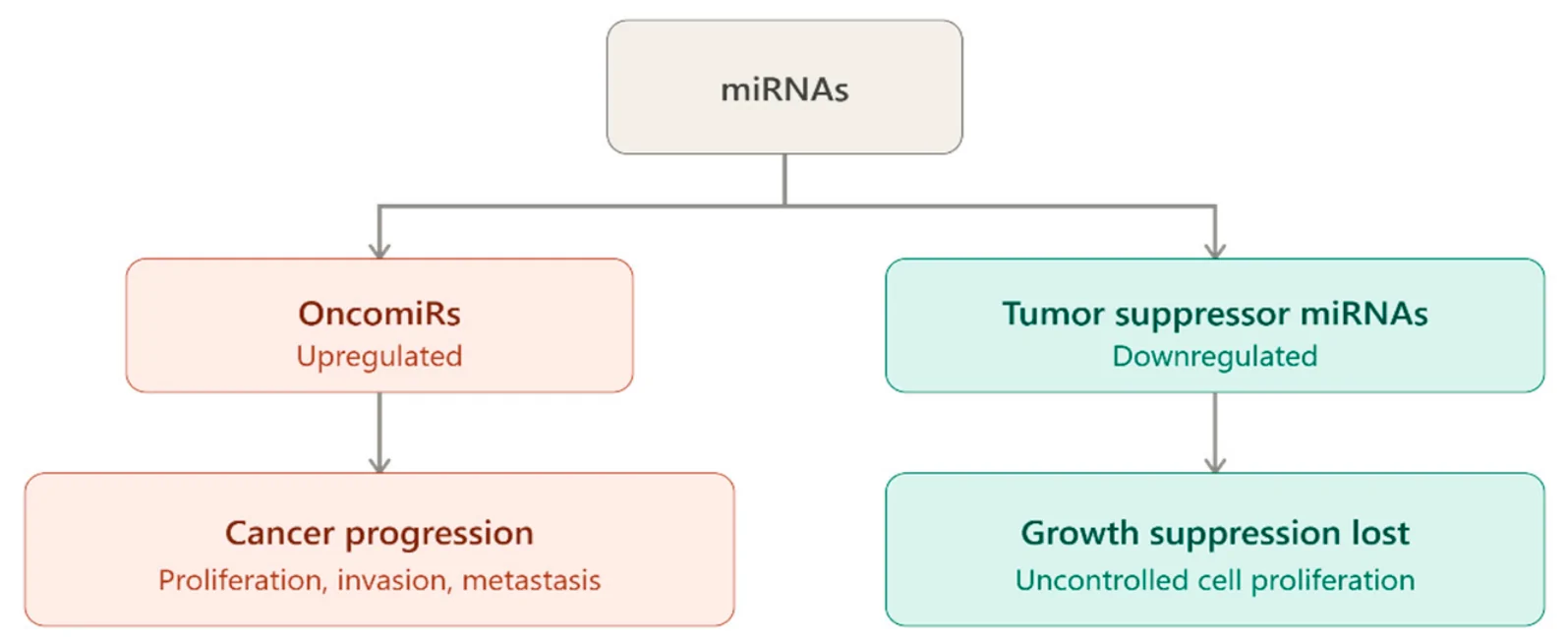
miRNAs classified according to their role as tumor suppressors or oncogenes (created with Claude Sonnet 5).

**Table 1:** Circulating miRNA biomarkers in cutaneous melanoma compared with other solid tumors.

Cancer Type	Representative Circulating miRNA(s)/Panel	Reported Diagnostic Performance	Reference
Cutaneous melanoma	MEL38 (38-miRNA panel)	Sensitivity 93%, specificity 98%, AUC 0.98 (MEL38 vs. benign nevi and other controls)	Van Laar et al., 2023, Br J Dermatol [15]
Breast cancer	5-miRNA panel (miR-1246, miR-1307-3p, miR-4634, miR-6861-5p, miR-6875-5p)	Sensitivity 97.3%, specificity 82.9%, AUC 0.971 (multi-institutional test cohort)	Shimomura et al., 2016, Cancer Sci [30]
Colorectal cancer	miR-92a	Sensitivity 89%, specificity 70%, AUC 0.885	Ng et al., 2009, Gut [31]
Pancreatic cancer	4-miRNA panel (miR-21, miR-210, miR-155, miR-196a)	Sensitivity 64%, specificity 89%, AUC 0.82	Wang et al., 2009, Cancer Prev Res [32]
Prostate cancer	miR-141	46-fold higher in metastatic disease vs. healthy controls (founding study)	Mitchell et al., 2008, PNAS [33]
Pan-cancer (miR-21 alone)	miR-21	Pooled sensitivity 78%, specificity 82%, AUC 0.87 across at least 15 tumor types (36 studies)	Wu, Li & Li, 2015, Tumour Biol [29]

**Table 2:** Circulating miRNA evidence for clinical utility, mapped to established comparators across melanoma care.

Domain	Circulating miRNA Evidence	Established Comparator	Assessment/Gap
Early detection	Pooled diagnostic sensitivity 86–91%, specificity 81–89% (meta-analyses); no study tests miRNA as a pre-biopsy triage adjunct [12,47]	Dermoscopy and clinical exam (Cochrane review): sensitivity ~92% at a fixed specificity of 80%, or specificity ~95% at a fixed sensitivity of 80% [48]	No head-to-head comparison exists; pooled miRNA accuracy is numerically at or below expert dermoscopy—no demonstrated incremental value shown to date
Diagnosis (vs. benign lesions)	MEL38 panel: sensitivity 93%, specificity 98%, AUC 0.98, tested against benign nevi and other controls [15] 3-miRNA panel AUC 0.84 vs. nevi specifically [49]	Dermoscopy, guided biopsy, and histopathology	Reasonably strong direct evidence for melanoma-vs-nevus discrimination
Prognosis beyond AJCC stage	MEL12 signature: high- vs. low-risk HR 7.69 (95% CI 2.27–28.08, *p* = 0.0011), adjusted for age, sex, systemic therapy, and disease extent; 10-year melanoma-specific survival: 94% (low) vs. 58% (high risk) [15] The 9-miRNA plasma panel discriminated stage IV from lower-stage disease in treatment-naive patients with AUC 0.739 [50]	AJCC staging alone	The MEL12 panel shows prognostic value that persists after adjustment for stage-related covariates The 9-miRNA panel shows stage discrimination but has not been tested prospectively as a prognostic marker; independent replication is needed for both
Recurrence/MRD	No study quantifies a lead time of miRNA detection over clinical/imaging surveillance	Imaging and clinical surveillance; circulating tumor cells (CTCs) have shown lead time over imaging in at least one stage III study	Explicit evidence gap—flagged as a priority for future prospective studies
Treatment response prediction	Pre-treatment serum miR-579-3p/miR-4488: AUC 0.62–0.70 for PFS on BRAF-targeted therapy [51]; miR-1246/miR-485-3p AUC > 0.80 [52]	RECIST-based imaging response assessment	Modest-to-moderate discrimination in small, single-center cohorts; hypothesis-generating rather than practice-ready
Treatment resistance detection	4-miRNA extracellular-vesicle signature distinguishing primary treatment resistance: AUC 0.80 (discovery)/0.94 (validation) (*n* = 18 discovery/8 validation) 2-miRNA pair in the same study flagged emerging (acquired) resistance by ~week 4 of therapy, before clinical progression [53]	Clinical/radiologic progression	Promising signal but from a very small cohort; requires validation in larger series before any claim of early detection
Disease monitoring vs. imaging	No study directly compares miRNA-based monitoring performance with CT/PET-CT/MRI surveillance	CT, PET-CT, MRI surveillance	Explicit evidence gap
Combination with ctDNA	Combined single-tube co-detection of miRNA, ctDNA, and CTCs is technically feasible [54]; no study reports the combined diagnostic/prognostic performance of miRNA + ctDNA against either marker alone	ctDNA alone	Explicit evidence gap; feasibility does not equal demonstrated added value
Clinical implementation barriers	Marked inter-study heterogeneity (I2 > 50%) attributable to specimen type, geography, and collection timing [12]; no circulating-miRNA assay for melanoma has regulatory or clinical approval to date	N/A	Standardization of collection, processing, and normalization is the rate-limiting barrier to translation, more than biological plausibility

**Table 3:** A summary of miRNA roles.

miRNA	Specimen Type	Evidence Category	Role/Notes
miR-21	Tissue/serum	Biological role (oncomiR)	Upregulated in melanoma; promotes cancer progression [28].
miR-221	Serum	Diagnostic/prognostic	Elevated serum levels in stage I–IV melanoma; correlates with tumor thickness; promotes progression by targeting tumor suppressor genes [28,34].
miR-221-3p	Serum	Diagnostic	Significantly upregulated in melanoma patients vs. healthy controls; promotes melanoma progression [12].
miR-34a	Tissue	Biological role (tumor suppressor)	Acts as tumor suppressor in melanoma [28].
hsa-miR-193a-3p	Tissue/cell line	Therapeutic target (preclinical)	Overexpression reduces cell viability; affects genes involved in proliferation and apoptosis [26].
hsa-miR-193a-5p	Tissue/cell line	Therapeutic target (preclinical)	Overexpression reduces cell viability; affects genes involved in proliferation and apoptosis [26].
miR-153-3p	Tissue/cell line	Therapeutic target (preclinical)	Inhibits cell proliferation and invasion by targeting SNAI1 [27].
miR-206	Serum	Prognostic	Decreased serum levels associated with poor prognosis, advanced clinical stage, and lower survival rates [35].
miR-125b	Serum exosome	Prognostic	Lower serum exosome levels linked with advanced melanoma [36].
miR-30d	Plasma	Prognostic	Part of a 4-miRNA serum signature (with miR-150, miR-15b, miR-425) that stratified recurrence-free and overall survival in a cohort of 283 patients, improving on AJCC stage alone [43].
miR-1246	Plasma	Diagnostic/monitoring	Associated with metastatic melanoma profile; high sensitivity and specificity as non-invasive monitoring biomarker [44].
miR-185	Plasma	Diagnostic/monitoring	Associated with metastatic melanoma; potential non-invasive monitoring biomarker [44].
miR-150-5p	Serum	Diagnostic	Potential diagnostic marker for stage IV melanoma; distinct expression vs. healthy individuals; present in melanoma cell lines [21].
miR-155-5p	Serum	Diagnostic/predictive	Diagnostic marker for stage IV melanoma. High baseline levels associated with better PFS and OS in patients on immune checkpoint inhibitors; linked to CD4+ T-cell subpopulations [21].
miR-16	Serum/plasma	Methodological (normalizer)	Reduced in melanoma patients vs. controls; commonly used as a reference normalizer in miRNA studies [38,39].
miR-155-5p/miR-424-5p/miR-320a	Plasma	Predictive	High baseline levels associated with longer progression-free survival (PFS) and overall survival (OS) in patients treated with immune checkpoint inhibitors [45].
MEL12 (12-miRNA signature)	Plasma	Prognostic	Stratifies patients into risk groups; correlates with clinical staging and sentinel lymph node biopsy. High-risk patients ~3× more likely to have melanoma detected in sentinel lymph nodes [15].
6-miRNA signature	Tissue	Prognostic	Independent predictor of post-recurrence survival in metastatic melanoma [46].
MEL38 (38-miRNA signature)	Plasma	Diagnostic	93% sensitivity, 98% specificity for invasive melanoma diagnosis; AUC of 0.98 [15].
MELmiR-7 panel	Serum/plasma	Monitoring/predictive	Predicts treatment outcomes and overall survival in stage IV melanoma patients undergoing therapy [57]

## Data Availability

No new data were created or analyzed in this study. Data sharing is not applicable to this article, all data discussed are available in the cited publications.

## Abbreviations

The following abbreviations are used in this manuscript:

AJCC	American Joint Committee on Cancer
CT	Computed tomography
PET-CT	Positron emission tomography–computed tomography
MRI	Magnetic resonance imaging
LDH	Lactate dehydrogenase
CTC	Circulating tumor cell
ctDNA	Circulating tumor DNA
miRNA	microRNA
miRISC	miRNA-induced silencing complex
ICI	Immune checkpoint inhibitor
PFS	Progression-free survival
OS	Overall survival
PD-1	Programmed cell death protein 1
CTLA-4	Cytotoxic T-lymphocyte-associated protein 4
AUC	Area under the (receiver operating characteristic) curve
qRT-PCR	Quantitative reverse transcription polymerase chain reaction
